# Epidemiology, risk factors and clinical course of SARS-CoV-2 infected patients in a Swiss university hospital: An observational retrospective study

**DOI:** 10.1371/journal.pone.0240781

**Published:** 2020-11-13

**Authors:** Jean Regina, Matthaios Papadimitriou-Olivgeris, Raphaël Burger, Marie-Annick Le Pogam, Tapio Niemi, Paraskevas Filippidis, Jonathan Tschopp, Florian Desgranges, Benjamin Viala, Eleftheria Kampouri, Laurence Rochat, David Haefliger, Mehdi Belkoniene, Carlos Fidalgo, Antonios Kritikos, Katia Jaton, Laurence Senn, Pierre-Alexandre Bart, Jean-Luc Pagani, Oriol Manuel, Loïc Lhopitallier

**Affiliations:** 1 Service of Infectious Diseases, Lausanne University Hospital and University of Lausanne, Lausanne, Switzerland; 2 Service of Hospital Preventive Medicine, Lausanne University Hospital and University of Lausanne, Lausanne, Switzerland; 3 Service of Internal Medicine, Lausanne University Hospital and University of Lausanne, Lausanne, Switzerland; 4 Centre for Primary Care and Public Health (Unisanté), University of Lausanne, Lausanne, Switzerland; 5 Institute of Microbiology, Lausanne University Hospital and University of Lausanne, Lausanne, Switzerland; 6 Service of Intensive Care, Lausanne University Hospital and University of Lausanne, Lausanne, Switzerland; Azienda Ospedaliero Universitaria Careggi, ITALY

## Abstract

**Background:**

This study aims to describe the epidemiology of COVID-19 patients in a Swiss university hospital.

**Methods:**

This retrospective observational study included all adult patients hospitalized with a laboratory confirmed SARS-CoV-2 infection from March 1 to March 25, 2020. We extracted data from electronic health records. The primary outcome was the need to mechanical ventilation at day 14. We used multivariate logistic regression to identify risk factors for mechanical ventilation. Follow-up was of at least 14 days.

**Results:**

145 patients were included in the multivariate model, of whom 36 (24.8%) needed mechanical ventilation at 14 days. The median time from symptoms onset to mechanical ventilation was 9·5 days (IQR 7.00, 12.75). Multivariable regression showed increased odds of mechanical ventilation with age (OR 1.09 per year, 95% CI 1.03–1.16, p = 0.002), in males (OR 6.99, 95% CI 1.68–29.03, p = 0.007), in patients who presented with a qSOFA score ≥2 (OR 7.24, 95% CI 1.64–32.03, p = 0.009), with bilateral infiltrate (OR 18.92, 3.94–98.23, p<0.001) or with a CRP of 40 mg/l or greater (OR 5.44, 1.18–25.25; p = 0.030) on admission. Patients with more than seven days of symptoms on admission had decreased odds of mechanical ventilation (0.087, 95% CI 0.02–0.38, p = 0.001).

**Conclusions:**

This study gives some insight in the epidemiology and clinical course of patients admitted in a European tertiary hospital with SARS-CoV-2 infection. Age, male sex, high qSOFA score, CRP of 40 mg/l or greater and a bilateral radiological infiltrate could help clinicians identify patients at high risk for mechanical ventilation.

## Introduction

Severe acute respiratory syndrome coronavirus 2 (SARS-CoV-2) first emerged in Wuhan (China) in December 2019. WHO, in February 2020, named the resulting disease COronaVirus Disease 2019 (COVID-19) [1]. There is a wide spectrum of severity ranging from asymptomatic presentation to severe pneumonia requiring ventilator support and death [2]. Since March 2020, the pace of SARS-CoV-2 spread around the globe increased as the epidemic evolved to a pandemic [3]. In Europe, following a diminished caseload during summer 2020, new cases and deaths are increasing since September 2020 [4].

In the present study, we report the epidemiological and clinical characteristics of patients hospitalized for COVID-19 in a Swiss university hospital during the early phase of the pandemic as well as risk factors for progressive respiratory failure requiring mechanical ventilation.

## Methods

### Study design and participants

In this retrospective observational study, we included all adult patients consecutively hospitalized with a confirmed SARS-CoV-2 infection from March 1, 2020, to March 25, 2020. Patient’s initial physicians used local guidelines to decide on admission (only patients with risk factors for severe disease or needing medical care were hospitalized). For all patients, we ensured a follow-up of 14 days or more during hospital stay or up to discharge or death if they occurred first.

### Study setting

This study took place in Lausanne University Hospital (LUH), a one-thousand-bed tertiary university hospital in Lausanne, Switzerland. LUH serves as a primary-level community hospital for Lausanne (population circa 300’000 inhabitants) and as a referral hospital for Western Switzerland (population circa 1–1.5 million inhabitants). LUH increased its outbreak response capacity by setting up new intensive care units for the management of COVID-19 patients.

### Data collection

LUH electronic health record (EHR) provided data on epidemiological, clinical, radiological and laboratory data.

Epidemiological data included age, sex, height, weight, and relevant comorbidities, including the Charlson Comorbidities Index (CCI). We collected data on clinical presentation, SARS-CoV-2 treatments, concomitant treatments, non-pharmacological interventions and clinical course within LUH.

We recorded radiological findings from reports of chest radiography or computed tomography (CT). We defined healthcare workers as professionals having direct contact with patients (nurses, physiotherapists, physicians, etc.) or patient samples.

Laboratory data included full blood count, D-dimers, creatinine, highly sensitive cardiac T-troponin, C-reactive protein (CRP), procalcitonin (PCT), ferritin, liver function tests, blood type and real-time PCR to detect SARS-CoV-2 [5].

We entered all data in an electronic clinical report form (eCRF) using the REDCap^®^ platform (Research Electronic Data Capture v8.5.24, Vanderbilt University, Tennessee, USA) [6]. Fellows of the Infectious Diseases, Hospital Preventive Medicine and Internal Medicine Services at LUH entered the data and two of the authors (JR, MP) verified their integrity.

### Clinical management

Treating physicians made all decisions regarding supportive care. Specialists in infectious diseases reviewed all SARS-CoV-2 treatment decisions according to the local recommendations. These included protease inhibitors (ritonavir-boosted lopinavir or atazanavir), hydroxychloroquine or remdesivir. Selected critically ill patients with high inflammatory markers (CRP, D-dimers, PCT and ferritin) received tocilizumab. The choice of treatment depended on drug availability, safety profile drug-drug interactions.

Treating physicians discussed *advance planning of care* and *do not resuscitate orders* with all patients. Limitations of care were decided upon admission according to the patient’s values and goals and the treating physician’s appreciation. Treating physicians documented all inpatients’ limitation of care in LUH EHR. We categorized these limitations into two levels: 1) no limitation; 2) limitation to the best supportive care provided in non-monitored wards or intermediate care units but without mechanical ventilation (MV).

### Definition

We defined a confirmed SARS-CoV-2 infection as a positive test for SARS-CoV-2 using real-time polymerase chain reaction (qPCR) technology in any respiratory sample.

We defined obesity either as a body mass index (BMI) of 30 kg/m^2^ or higher, or, when missing anthropometric data, a medical diagnosis of obesity.

We used the Berlin definition for Acute Respiratory Distress Syndrome (ARDS) [7]. We defined *shock* as refractory hypotension requiring infusion of vasopressors. Acute kidney injury (AKI) was identified and classified according to the 2012 Kidney Disease Improving Global Outcome guidelines [8]. We defined liver injury as a 3-fold or greater increase in transaminase levels.

We calculated quick Sequential Organ Failure Assessment (qSOFA) score, Confusion/Respiratory rate/Blood pressure/age ≥ 65 years (CRB-65) score and National Early Warning Score (NEWS) were assessed according to their original descriptions [9–11].

We defined MV as invasive respiratory support through a laryngeal or a tracheostomy tube

### Outcome

The primary outcome was the use of MV for respiratory failure attributed to SARS-CoV-2 pneumonia, within 14 days after admission.

### Statistics

For the descriptive analysis, we first described patient’s characteristics. We summarized categorical variables as numbers (percentages), normally distributed continuous variables as mean ± standard deviation (SD) and continuous variables with a skewed distribution as median [interquartile range (IQR)]. We then tested for associations between patient’s characteristics and MV. We used Pearson’s chi-square test for binary characteristics, Student t-test for normally distributed variables and Mann-Whitney-Wilcoxon test for continuous variables with a skewed distribution.

We used a general linear model based on univariate logistic regression to calculate odds ratio for MV. We finally performed a multivariate logistic regression with a LASSO (Least Absolute Shrinkage and Selection Operator) penalisation. We used LASSO regression analysis, as the number of MV cases was low and did not allow us to obtain a multivariate regression model with more than four predictors. LASSO provides the most parsimonious model for high number of covariates and small sample size [12]. In addition, it accounts for collinearity. For multivariate analysis, we selected relevant clinical, biological and radiological parameters as well as patients characteristics previously identified in the literature and after authors consensus (clinical knowledge). To identify the optimal penalization parameter (lambda), we used a bootstrap resampling procedure: 1000 bootstrap samples of the same size of the full database (lambda = 4). We retained as the model the one with the lowest AIC (Akaike Information Criterion).

We excluded from the analysis, patients whose care was limited to the best supportive care and patients already mechanically ventilated on admission. As we did not have treatments starting date, we did not include SARS-CoV-2 specific treatments as variables in the model.

For the inflammatory biomarkers, we converted continuous variables to categorical variables using cut-off values previously identified in the literature: 1000 ng/ml for D-dimer, 40 mg/L for CRP, 0.5 μg/l for procalcitonin and 300 μg/l for ferritin [13–15].

We did not impute any values for missing data.

Statistical analyses were performed using R software v3.6.2 (R Foundation for Statistical Computing; www.r-project.org).

### Ethics

This project was conducted in accordance with the Declaration of Helsinki, the principles of Good Clinical Practice and the Swiss Human Research Act (HRA). The project received approval from the Ethics Committee of canton Vaud, Switzerland (2020–00657) that waived the need for informed consent. All data were anonymized before analysis.

## Results

### Epidemiological characteristics

Overall, 200 patients with confirmed SARS-CoV-2 infection were hospitalized at LUH during the study period. In 54 (28.5%), care was agreed to be limited to best supportive care on admission, these patients were older and with more comorbidities (Table 1).

**Table 1:** Comparison of patients with and without limitation of care.

	No limitation of care	Limitation of care	*P* Value
n	146	54	
Age, years (median [IQR])	62.50 [52.00, 74.00]	83.50 [80.00, 88.00]	<0.001
Age group, years (n, %)			<0.001
18–30	7 (4.8)	1 (1.9)	
31–50	28 (19.2)	0 (0.0)	
51–65	47 (32.2)	3 (5.6)	
65–80	48 (32.9)	11 (20.4)	
>80	16 (11.0)	39 (72.2)	
Charlson Comorbidity Index (median [IQR])	3.00 [1.00, 5.00]	7.00 [5.00, 9.00]	<0.001
Dementia (n, %)	5 (3.4)	21 (38.9)	<0.001
Cancer (n, %)	16 (11.0)	10 (18.5)	0.24

**Fig 1:**
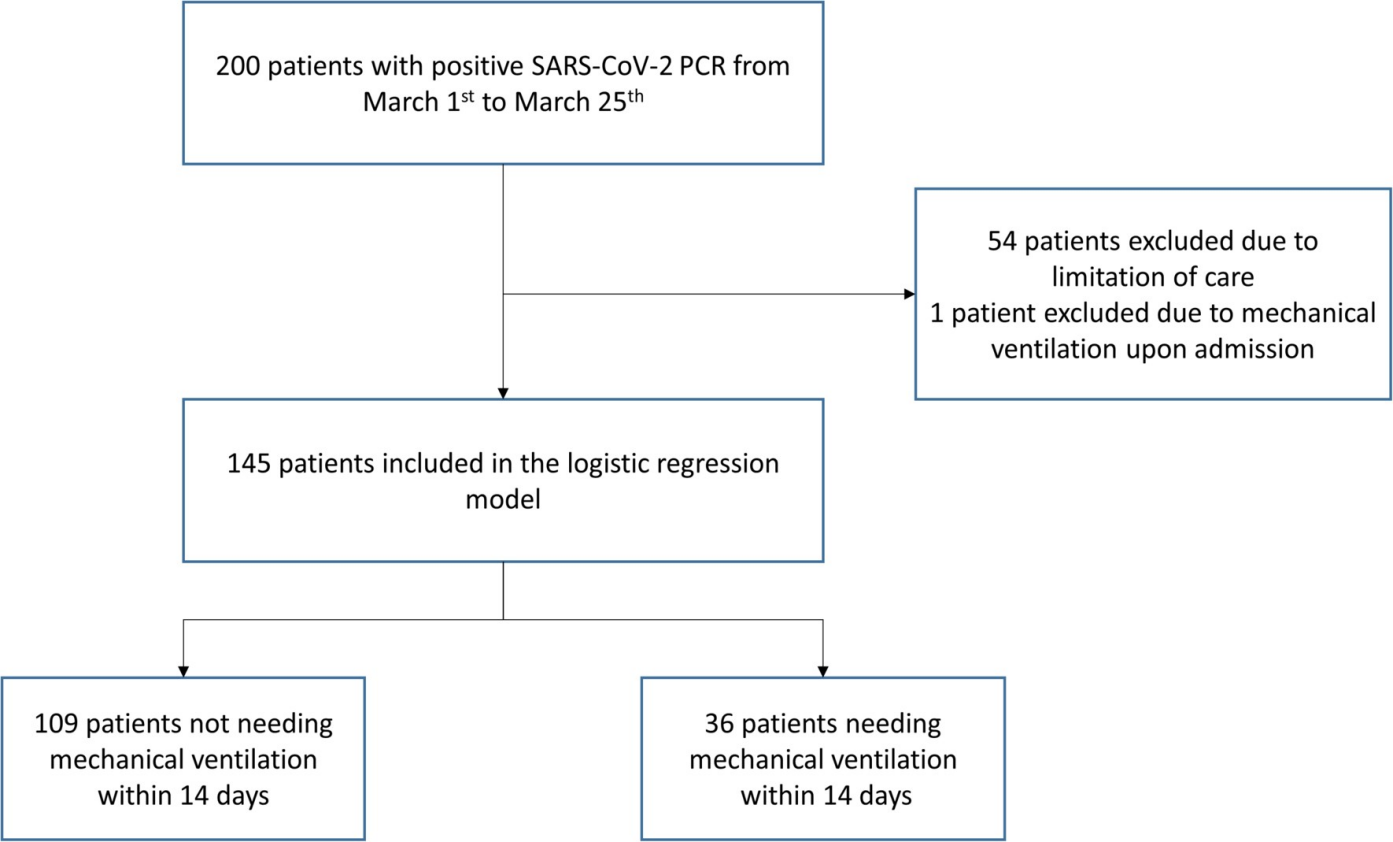
Patient’s selection for univariate and multivariate models.

We excluded 54 patients with limitation of care and one patient already mechanically ventilated of admission and included 145 (72.5%) patients in the statistical analysis (Fig 1).

Median patient age was 62.0 years [IQR 52.0–74.0], ranging from 20.0 to 89.0 years. Eighty-one (55.8%) of all inpatients were aged 65.0 years or younger. Median BMI was 26.99 [IQR 23.84–31.84].

One hundred and nine (76.2%) patients had at least one comorbidity, with a median Charlson Comorbidity Index of 3.0 [IQR 1.0–5.0]. Table 2 summarizes the characteristics of this population whose most frequent comorbidities were hypertension (39.3%), obesity (33.8%), diabetes (21.4%), coronary artery disease (13.1%) and chronic kidney disease (10.3%).

**Table 2:** Demographics of patients on admission.

	Overall	No Mechanical Ventilation	Mechanical Ventilation	*P* Value
n	145	109	36	
Male sex (n, %)	90 (62.1)	62 (56.9)	28 (77.8)	0.041
Age, years (median [IQR])	62.00 [52.00, 74.00]	62.00 [50.00, 73.00]	68.00 [56.75, 79.25]	0.069
Age group, years (n, %)				0.399
18–30	7 (4.8)	7 (6.4)	0 (0.0)	
31–50	28 (19.3)	21 (19.3)	7 (19.4)	
1–65	46 (31.7)	36 (33.0)	10 (27.8)	
65–80	48 (33.1)	35 (32.1)	13 (36.1)	
>80	16 (11.0)	10 (9.2)	6 (16.7)	
Overweight (BMI>25) (n, %)	82 (56.6)	56 (51.4)	26 (72.2)	0.046
Obesity (BMI>30) (n, %)	49 (33.8)	33 (30.3)	16 (44.4)	0.175
Body mass index (median [IQR])	26.99 [23.84, 31.84]	26.83 [22.94, 32.04]	27.10 [24.22, 30.82]	0.802
Pregnant (n, %)	6 (10.9)	5 (10.6)	1 (12.5)	1
Health care worker (n, %)	6 (4.2)	4 (3.7)	2 (5.6)	1
Comorbidity (n, %)	109 (76.2)	83 (77.6)	26 (72.2)	0.67
Hypertension (n, %)	57 (39.3)	42 (38.5)	15 (41.7)	0.891
Atrial fibrillation (n, %)	7 (4.8)	5 (4.6)	2 (5.6)	1
Coronary artery disease (n, %)	19 (13.1)	13 (11.9)	6 (16.7)	0.656
Stroke (n, %)	7 (4.8)	5 (4.6)	2 (5.6)	1
Chronic kidney disease (n, %)	15 (10.3)	13 (11.9)	2 (5.6)	0.44
Chronic obstructive pulmonary disease (n, %)	10 (6.9)	6 (5.5)	4 (11.1)	0.44
Asthma (n, %)	7 (4.8)	6 (5.5)	1 (2.8)	0.831
Diabetes (n, %)	31 (21.4)	23 (21.1)	8 (22.2)	1
Cirrhosis (n, %)	5 (3.4)	5 (4.6)	0 (0.0)	0.435
Charlson Comorbidity Index (median [IQR])	3.00 [1.00, 5.00]	3.00 [1.00, 5.00]	4.00 [1.00, 5.00]	0.312
Transplantation (n, %)	6 (4.1)	6 (5.5)	0 (0.0)	0.339
Hematopoietic stem cell transplantation (n, %)	1 (0.7)	1 (0.9)	0 (0.0)	0.339
Solid organ transplantation (n, %)	5 (3.4)	5 (4.6)	0 (0.0)	0.435
Cancer (n, %)	16 (11.0)	13 (11.9)	3 (8.3)	0.772
HIV infection (n, %)	3 (2.1)	2 (1.8)	1 (2.8)	1
Immunosupressive drugs (n, %)	9 (6.2)	9 (8.3)	0 (0.0)	0.167
ACE inhibitors or ARB II (n, %)	31 (22.0)	20 (18.7)	11 (32.4)	0.15
NSAIDs (n, %)	2 (1.4)	2 (1.9)	0 (0.0)	1

Abbreviations: BMI, body mass index; HIV, human immunodeficiency virus; ACE, angiotensin converting enzyme; ARB, angiotensin receptor blocker; NSAIDs, nonsteroidal anti-inflammatory drugs.

Thirty-one (22.0%) patients were treated with angiotensin-converting enzyme inhibitors (ACEI) or angiotensin II receptor blockers (ARBs). Nine (6.2%) were treated with immunosuppressive drugs of which six (4.1%) patients were transplant recipients (5 solid-organ and 1 haematopoietic stem-cell transplantation). Sixteen (11.0%) patients had an active malignancy at the time of admission.

### Clinical characteristics on admission

Table 3 describes clinical characteristics of patients on admission. The mean duration of symptoms preceding SARS-CoV-2 RT-PCR test was seven days [IQR 4.0–10.0]. The most frequent symptoms at the time of testing were fever in 104 patients (71.7%), cough in 100 (69.0%), fatigue in 72 (49.7%) and dyspnoea in 77 (53.1%).

**Table 3:** Clinical characteristics and radiology of patients on admission.

	Overall	No Mechanical Ventilation	Mechanical Ventilation	*P* Value
n	145	109	36	
Duration of symptoms before test, days (mean(SD))	7.00 [4.00, 10.00]	7.00 [3.00, 9.00]	6.50 [4.75, 10.25]	0.348
Fever (n, %)	104 (71.7)	78 (71.6)	26 (72.2)	1
Fatigue (n, %)	72 (49.7)	54 (49.5)	18 (50.0)	1
Cough (n, %)	100 (69.0)	71 (65.1)	29 (80.6)	0.127
Anosmia/Dysgeusia (n, %)	6 (4.1)	5 (4.6)	1 (2.8)	1
Dyspnoea (n, %)	77 (53.1)	49 (45.0)	28 (77.8)	0.001
Arthralgia/Myalgia (n, %)	36 (24.8)	26 (23.9)	10 (27.8)	0.803
Nausea/Vomitting (n, %)	34 (23.4)	30 (27.5)	4 (11.1)	0.074
Diarrhea (n, %)	35 (24.1)	30 (27.5)	5 (13.9)	0.152
Loss of consciousness (n, %)	13 (9.0)	9 (8.3)	4 (11.1)	0.855
Confusion (n, %)	7 (4.8)	3 (2.8)	4 (11.1)	0.114
Temperature (median [IQR])	38.30 [37.70, 38.80]	38.20 [37.65, 38.70]	38.70 [37.98, 39.12]	0.024
Systolic blood pressure (median [IQR])	117.00 [105.00, 127.00]	118.00 [109.00, 129.00]	109.50 [97.00, 125.00]	0.03
Heart rate (median [IQR])	93.00 [85.00, 104.50]	93.00 [83.50, 104.00]	94.50 [88.00, 115.25]	0.087
Respiratory rate (median [IQR])	24.50 [20.00, 30.00]	22.00 [18.25, 28.00]	30.50 [27.75, 35.00]	<0.001
Glasgow Coma Scale = 15 (n, %)	15.00 [15.00, 15.00]	15.00 [15.00, 15.00]	15.00 [15.00, 15.00]	0.003
Oxygen, liters per minute (median [IQR])	2.00 [0.00, 3.00]	1.00 [0.00, 2.00]	4.50 [2.00, 8.50]	<0.001
Oxygen saturation, percent (median [IQR])	97.00 [95.00, 98.00]	97.00 [95.50, 98.00]	96.00 [93.00, 97.00]	0.019
Co-infection on admission (n, %)	39 (26.9)	20 (18.3)	19 (52.8)	<0.001
Chest X-ray (n, %)	135 (93.1)	99 (90.8)	36(100.0)	0.133
Lung scan (n, %)	15 (10.3)	9 (8.3)	6/36 (16.7)	0.262
Radiological infiltrate (n, %)	100 (69.0)	66 (60.6)	34/36 (94.4)	<0.001
Bilateral (n, %)	82 (56.6)	51 (46.8)	31/36 (86.1)	<0.001
CRB-65 score				<0.001*
0–1 (n, %)	100/139 (71.9)	83/103 (80.6)	17/36 (47.2)	
2 = 1 (n, %)	25/139 (18.0)	14/103 (13.6)	11/36 (30.6)	
3–5 = 1 (n, %)	14/139 (10.1)	6/103 (5.8)	8/36 (22.2)	
qSOFA score	142 (97.9)	106 (97.2)	36 (100.0)	<0.001*
0 (n, %)	54/142 (38.0)	51/106 (48.1)	3/36 (8.3)	
1 (n, %)	65/142 (45.8)	46/106 (43.4)	19/36 (52.8)	
2 (n, %)	19/142 (13.4)	8/106 (7.5)	11/36 (30.6)	
3 (n, %)	4/142 (2.8)	1/106 (0.9)	3/36 (8.3)	
NEWS score	138 (95.2)	106 (97.2)	32 (88.9)	<0.001*
0 (n, %)	7/138 (5.1)	7/106 (6.6)	0/32 (0.0)	
1–4 (n, %)	40/138 (29.0)	38/106 (35.8)	2/32 (6.2)	
5–6 (n, %)	31/138 (22.5)	27/106 (25.5)	4/32 (12.5)	
7 or more (n, %)	60/138 (43.5)	34/106 (32.1)	26/32 (81.2)	

CRB-65 score, Confusion/Respiratory rate/Blood pressure/age ≥ 65 years score; qSOFA score, quick Sequential Organ Failure Assessment score; NEWS, National Early Warning Score

*χ^2^ test comparing all subcategories.

Overall, 100 (69.0%) patients had a radiological exam and presented new lung infiltrates which were bilateral for 82 (56.6%) of them. Table 3 describes vital signs, qSOFA score, NEWS and CRB-65 scores on admission.

### Laboratory values

Table 4 describes the median value of commonly measured inflammatory parameters (white blood cell count, CRP, procalcitonin, D-dimers and ferritin).

**Table 4:** Laboratory values of patients on admission.

	Overall	No Mechanical Ventilation	Mechanical Ventilation	*P* Value
n	145	109	36	
White blood cell count, x109 per L (median [IQR])	5.80 [4.40, 7.30]	5.30 [4.30, 6.70]	6.45 [5.07, 10.12]	0.005
Lymphocytes count, x109 per L (median [IQR])	0.85 [0.62, 1.23]	0.85 [0.62, 1.29]	0.86 [0.63, 1.15]	0.828
Platelets count, x109 per L (median [IQR])	202.00 [154.00, 256.00]	212.00 [160.00, 270.00]	187.50 [144.00, 238.00]	0.133
D-dimer, ng/mL (median [IQR])	884.00 [532.75, 1696.00]	782.00 [462.00, 1567.00]	1155.00 [706.00, 2241.00]	0.02
Creatinine, μmol/L (median [IQR])	87.00 [72.50, 112.00]	84.00 [70.00, 110.00]	94.00 [81.75, 124.75]	0.082
AKIN classification (n, %)				0.003*
0	105 (75.0)	86 (82.7)	19 (52.8)	
1	29 (20.7)	16 (15.4)	13 (36.1)	
2	3 (2.1)	1 (1.0)	2 (5.6)	
3	3 (2.1)	1 (1.0)	2 (5.6)	
CRP, mg/L (median [IQR])	54.50 [26.25, 119.00]	43.00 [18.00, 85.75]	127.50 [55.75, 189.00]	<0.001
PCT, μg/L (median [IQR])	0.15 [0.08, 0.25]	0.11 [0.07, 0.19]	0.21 [0.15, 0.46]	<0.001
Serum ferritin, μg/L (median [IQR])	1030.00 [544.00, 1648.00]	857.00 [517.00, 1485.00]	1545.50 [747.00, 1901.75]	0.047
High-sensitive cardiac troponin I, ng/mL (median [IQR])	9.00 [6.00, 20.00]	9.00 [5.00, 17.00]	14.50 [7.75, 29.75]	0.015
AST, U/L (median [IQR])	44.50 [35.00, 67.50]	44.00 [34.00, 59.75]	66.00 [37.00, 88.25]	0.024
ALT, U/L (median [IQR])	33.00 [20.00, 58.00]	32.00 [20.00, 53.00]	39.00 [21.50, 65.25]	0.175
Total bilirubin, μmol/L (median [IQR])	8.00 [5.00, 12.00]	7.00 [5.00, 11.00]	9.00 [6.00, 14.00]	0.13
ABO group (n, %)				0.416*
A	36/88 (40.9)	26/56 (46.4)	10/32 (31.2)	
B	11/88 (12.5)	5/56 (8.9)	6/32 (18.8)	
AB	5/88 (5.7)	3/56 (5.4)	2/32 (6.2)	
O	36/88 (40.9)	22/56 (39.3)	14/32 (43.8)	

AKIN, Acute Kidney Injury Network; CRP, C-reactive protein; PCT, procalcitonin; AST, aspartate transaminase; ALT, alanine transaminase.

*χ^2^ test comparing all subcategories.

### Treatments

97 (66.9%) of all patients received SARS-CoV-2 treatment (Table 5). The most frequently prescribed medication were protease inhibitors in 86 patients (59.3%) and hydroxychloroquine in 70 patients (48.3%). Sixty-seven patients (46.2%) received two or more concomitant SARS-CoV-2 treatments. Fifty-six (38.6%) patients received antibiotics. One hundred and five (72.4%) patients required supplementary oxygen during the follow-up period.

**Table 5:** Treatments received by patients during the follow-up period.

	Overall	No mechanical ventilation	Mechanical ventilation	*P* Value
n	145	109	36	
Any SARS-CoV-2 treatment (n, %)	97 (66.9)	63 (57.8)	34 (94.4)	<0.001
Protease inhibitor (n, %)	86 (59.3)	55 (50.5)	31 (86.1)	<0.001
Hydroxychloroquine (n, %)	70 (48.3)	45 (41.3)	25 (69.4)	0.006
Remdesivir (n, %)	16 (11.0)	0 (0.0)	16 (44.4)	<0.001
Tocilizumab (n, %)	17 (11.7)	3 (2.8)	14 (38.9)	<0.001
Any antibiotic treatment (n, %)	56 (38.6)	25 (22.9)	31 (86.1)	<0.001

### Clinical course

Table 6 summarizes patient’s clinical course. At the end of the follow-up, 27 (18.6%) patients were still hospitalized, 87 (60.0%) patients were discharged, 8 (5.5%) patients were transferred to a rehabilitation centre and 9 (6.2%) to another acute care hospital. Fourteen (9.7%) patients died during hospitalization.

**Table 6:** Clinical course.

	Overall
N	145
Time from hospitalization to oxygen need, days* (median [IQR])	0.00 [0.00, 1.00]
Time from symptoms onset to mechanical ventilation, days (median [IQR])	9.50 [7.00, 12.75]
Time from hospitalization to mechanical ventilation, days* (median [IQR])	2.00 [0.00, 3.00]
Duration of mechanical ventilation of extubated patients, days (median [IQR])	6.00 [5.00, 11.00]
Outcome	
Still Hospitalized (n, %)	27 (18.6)
Discharge at home (n, %)	87 (60.0)
Time from hospitalization to home discharge, days* (median [IQR])	6.00 [4.00, 9.00]
Readaptation (n, %)	8 (5.5)
Time from hospitalization to readaptation discharge, days* (median [IQR])	17.00 [9.00, 18.00]
Transfer at other acute care hospital (n, %)	9 (6.2)
Time from hospitalization to other acute care hospital transfer, days* (median [IQR])	6.00 [6.00, 9.00]
Death (n, %)	14 (9.7)
Time from hospitalization to death, days* (median [IQR])	8.00 [4.00, 12.00]

Overall, 36 (24.8%) patients required mechanical ventilation after a median of two days since admission [IQR 0.00–3.00]. Median time from symptom onset to mechanical ventilation was 9.5 days [IQR 7.00–12.75]. Regarding patients requiring MV, 26 (72.2%) had a least one session of prone positioning, 24 (66.7%) received a vasopressor, 11 (30.5%) were eventually weaned from ventilator support and 11 (30.5%) died. The median duration of MV was six days [IQR 5.00–11.00]. Twenty-two of the 36 patients (61.1%) requiring MW were admitted to new dedicated COVID-19 ICUs.

### Complications

Table 7 describes complications during follow-up. The most frequent complications were ARDS (*n* = 41, 28.3%), acute kidney injury (*n* = 23, 15.9%), hospital-acquired pneumonia (HAP) (*n* = 21, 14.5%), acute confusional state (*n* = 16, 11.0%) and rhythm disorder (*n* = 16, 11.0%).

**Table 7:** Complications during the follow-up period.

	Overall	No mechanical ventilation	Mechanical ventilation	*P* Value
n	145	109	36	
Asthma or COPD exacerbation (n, %)	4 (2.8)	4 (3.7)	0 (0.0)	0.563
Community acquired pneumonia (n, %)	8 (5.5)	2 (1.8)	6 (16.7)	0.003
Hospital acquired pneumonia (n, %)	21 (14.5)	5 (4.6)	16 (44.4)	<0.001
Acute respiratory distress syndrome (n, %)	41 (28.3)	7 (6.4)	34 (94.4)	<0.001
Pneumothorax (n, %)	2 (1.4)	0 (0.0)	2 (5.6)	0.098
Pulmonary embolism (n, %)	5 (3.4)	3 (2.8)	2 (5.6)	0.785
Other thromboembolic event (n, %)	4 (2.8)	1 (0.9)	3 (8.3)	0.077
Acute confusional state (n, %)	16 (11.0)	9 (8.3)	7 (19.4)	0.121
Epileptic seizure (n, %)	1 (0.7)	0 (0.0)	1 (2.8)	0.559
Stroke (n, %)	3 (2.1)	1 (0.9)	2 (5.6)	0.308
Rythm disorder (n, %)	16 (11.0)	8 (7.3)	8 (22.2)	0.03
Myocarditis (n, %)	1 (0.7)	0 (0.0)	1 (2.8)	0.559
Heart failure (n, %)	2 (1.4)	1 (0.9)	1 (2.8)	0.995
Acute kidney injury (n, %)	23 (15.9)	6 (5.5)	17 (47.2)	<0.001
Septic shock (n, %)	12 (8.3)	1 (0.9)	11 (30.6)	<0.001
Acute hepatic injury (n, %)	10 (6.9)	5 (4.6)	5 (13.9)	0.126
Acute coronary syndrome (n, %)	2 (1.4)	0 (0.0)	2 (5.6)	0.098

Abbreviations: COPD: chronic obstructive pulmonary disease.

### Risk factors for mechanical ventilation

S1 Table summarizes unadjusted odds of MV at 14 days. Unadjusted odds of MV at 14 days were greater in males (odds ratio 2.65, 95% confidence interval 1.15 to 6.72) and in overweight patients (odds ratio 2.46, 95% confidence interval 1.11 to 5.81). None of the comorbidities increased the unadjusted risk MV at 14 days. Unadjusted odds of MV at 14 days were greater for patients presenting with dyspnoea (odds ratio 4.29, 95% confidence interval 1.86 to 10.85) on admission.

NEWS score ≥7 (odds ratio 9.18, 95% confidence interval 3.66 to 26.55), qSOFA score ≥2 (odds ratio 6.86, 95% confidence interval 2.68 to 18.47) or a CRB-65 score ≥2 (odds ratio 4.64, 95% confidence interval 2.07 to 10.65) increased the unadjusted odds of MV.

The presence of a radiological infiltrates increased the odds of mechanical ventilation (odds ratio 11.0, 95% confidence interval 3.14 to 70.45) as did the presence of a bilateral infiltrates (odds ratio 7.05, 95% confidence interval 2.75 to 21.87). Acute kidney injury on admission (odds ratio 4.27, 95% confidence interval 1.87 to 9.91), D-dimers of 1000 ng/ml or greater (odds ratio 3.28, 95% confidence interval 1.37 to 8.25), CRP of 40 mg/l or greater (odds ratio 6.79, 95% confidence interval 1.51 to 18.58) and PCT of 0.5 μg/l or greater (odds ratio 5.99, 95% confidence interval 1.52 to 29.66) increased the unadjusted odds of mechanical ventilation.

Table 8 summarizes adjusted odds of MV for patients with complete dataset. Multivariable regression showed increased odds of mechanical ventilation with age (OR 1.09 per year, 95% CI 1.03–1.16, p = 0.002), in males (OR 6.99, 95% CI 1.68–29.03, p = 0.007), in patients who presented with a qSOFA score ≥2 (OR 7.24, 95% CI 1.64–32.03, p = 0.009), with bilateral infiltrate (OR 18.92, 3.94–98.23, p<0.001) or with a CRP of 40 mg/l or greater (OR 5.44, 1.18–25.25; p = 0.030) on admission. Patients with more than seven days of symptoms on admission had decreased odds of mechanical ventilation (0.087, 95% CI 0.02–0.38, p = 0.001).

**Table 8:** Adjusted risk factors associated with mechanical ventilation at 14 days.

	Overall	No mechanical ventilation	Mechanical ventilation	Univariate OR [95% CI]	*P* value
n (%)	145	109	36		
Age (years)				1.09 [1.03, 1.16]	0.002
Male sex (%)	91 (62.3)	62 (56.9)	28 (77.8)	6.99 [1.68, 29.03]	0.007
Hypertension (%)	57 (39.0)	42 (38.5)	15 (41.7)	0.27 [0.07, 1.09]	0.066
Chronic obstructive pulmonary disease (%)	10 (6.8)	6 (5.5)	4 (11.1)	2.52 [0.35, 17.81]	0.354
More than seven days of symptoms (%)	80 (55.6)	62 (57.9)	18 (50.0)	0.087 [0.02, 0.38]	0.001
Dyspnea (%)	78 (53.4)	49 (45.0)	28 (77.8)	2.56 [0.65, 10.04]	0.178
Temperature > 38.2°C (%)	81 (56.6)	56 (52.3)	25 (69.4)	2.87 [0.80, 10.26]	0.104
Heart rate > 100 bpm (%)	48 (33.6)	32 (29.9)	16 (44.4)	2.71 [0.72, 10.17]	0.138
qSOFA score ≥ 2 (%)	23 (16.1)	9 (8.5)	14 (38.9)	7.24 [1.64, 32.03]	0.009
Bilateral radiological infiltrate (%)	83 (56.8)	51 (46.8)	31 (86.1)	18.92 [3.64, 98.23]	<0.001
Acute kidney injury on admission (%)	49 (45.0)	18 (17.3)	17 (47.2)	1.68 [0.50, 5.72]	0.403
C-reactive protein ≥ 40 mg/L (%)	86 (63.7)	53 (54.1)	32 (88.9)	5.44 [1.18, 25.25]	0.030

## Discussion

Our study identified several risk factors for unfavourable disease progression leading to MV in patients admitted with COVID-19 to a Swiss university hospital.

A quarter of the patients for which there were no limitation of care eventually required MV. MV occurred early during the course of hospitalization and the median duration of MV was shorter than previously reported [16, 17]. This effect is likely due to the limited duration of follow-up and could also result from a selection bias towards patients without limitations of care.

As infection with SARS-CoV-2 may cause an excessive host immune response, leading to ARDS and death [18], we would expect biomarkers of inflammation to be associated with unfavourable outcomes. In this study, CRP >40 mg/L on admission was associated with higher odds of MV, suggesting that an unfavourable course is more frequent in patients with a severe inflammatory response. Several studies have identified an increased risk of mortality in COVID-19 patients with elevated CRP [15, 19]. We believe that CRP is an ubiquitously measured biomarker whose result could potentially help clinicians assess the risk of MV in patients with COVID-19. Its use could be easily scaled up, and it is available as a point-of-care test.

In our study, the risk of mechanical ventilation increased with higher score values for NEWS, CRB-65 and qSOFA. qSOFA has been proven a useful predictor of mortality among patients with suspected infection [9], mainly of bacterial aetiology [20], but also influenza [21, 22]. We opted for including qSOFA in our final multivariate analysis, since it is widely used by clinicians in our institution, and is quick and easy to apply. A higher Sequential Organ Failure Assessment score (SOFA) has been previously linked to increased mortality due to COVID-19 [13]. Data for its calculation are not routinely collected for all patients outside the ICU, making it a less pragmatic tool to quickly evaluate the risk of MV in this patient population.

Age as a categorical variable was not significantly associated with MV in our univariate model but was significantly associated with MV when included as a continuous variable in our multivariate model. Numerous studies have linked age to mortality and to MV for SARS-CoV-2 patients [2, 13, 23]. The lack of significant association in the univariate model could be due to the small size of our sample and to limitations of care agreed to in older patients.

We additionally identify male sex as a predictor of unfavourable outcome in patients with COVID-19, as previously described [24–26]. A study recently underlined different immune response in male and female SARS-CoV-2 patients, which could explain more severe evolution in male patients [27].

Several factors such as obesity, pregnancy, healthcare worker status, previous ACE inhibitors or ARB II treatment and immunosuppressive drugs before admission were not associated with severe disease in this study.

Our study has several limitations to be acknowledged. First, due to its very nature the sample size is limited, which could lead to observation bias in the analyses, with some findings likely to evolve over time. The follow-up period was also limited and several patients were still hospitalised at the time of data analysis. Finally, due to the constantly evolving nature of the epidemic, the clinical care of patients likely evolved during their hospitalisation as changes were made to the recommendations for treatments administered at LUH. The single centre nature of the study limits the generalisability of the results.

However, this study gives some insights in the epidemiology and clinical course of patients admitted in our institution with SARS-CoV-2 infection. We found that age, male sex, bilateral SARS-CoV-2 pneumonia, elevated CRP and qSOFA equal or greater to two increased the risk of mechanical ventilation. The timely identification of these patients could help us target treatment and better manage the attribution of resources.

## Supporting information

S1 Table Unadjusted risk factors associated with mechanical ventilation at 14 days. (DOCX)

## Abbreviations

CCI: Charlson Comorbidity Index
AKI: Acute Kidney Injury
ARDS: Acute Respiratory Distress Syndrome
LUH: Lausanne University Hospital
EHR: Electronic Health Records
CRP: C-Reactive Protein
PCT: Procalcitonin
MV: Mechanical ventilation
SOFA: score Sequential Organ Failure Assessment score
qSOFA score: quick SOFA score
NEWS: National Early Warning score
CRB-65 score: Confusion/Respiratory rate/Blood pressure/age ≥ 65 years score
WHO: World Health Organization
