# Supplementary material for: Epidemiology, risk factors and clinical course of SARS-CoV-2 infected patients in a Swiss university hospital: An observational retrospective study
Source: PLoS One. 2020 Nov 13;15(11):e0240781. doi: 10.1371/journal.pone.0240781 (PMC7665644; doi:10.1371/journal.pone.0240781)
Supplement: S1 Table — (DOCX) [file pone.0240781.s001.docx]

|  | **Overall** | **No mechanical  ventilation** | **Mechanical  ventilation** | **Univariate OR  [95% CI]** | ***P* value** |
| --- | --- | --- | --- | --- | --- |
| n (%) | 145 | 109 | 36 |  |  |
| Age > 65 years (%) | 67 (45.9) | 48 (44.0) | 19 (52.8) | 1.42  [0.67, 3.05] | 0.363 |
| Male sex (%) | 91 (62.3) | 62 (56.9) | 28 (77.8) | 2.65 [1.15, 6.72] | 0.028 |
| Overweight (BMI>25 kg/m^2^) (%) | 83 (56.8) | 56 (51.4) | 26 (72.2) | 2.46  [1.11, 5.81] | 0.031 |
| Obesity (BMI>30 kg/m^2^) (%) | 49 (33.6) | 33 (30.3) | 16 (44.4) | 1.84  [0.84, 4.00 | 0.122 |
| Pregnancy (%) | 6 (10.9) | 5 (10.6) | 1 (12.5) | 1.2  [0.06, 9.08] | 0.876 |
| Hypertension (%) | 57 (39.0) | 42 (38.5) | 15 (41.7) | 1.14  [0.52, 2.44 | 0.739 |
| Coronary artery disease (%) | 19 (13.0) | 13 (11.9) | 6 (16.7) | 1.48  [0.48, 4.09 | 0.467 |
| Diabetes (%) | 31 (21.2) | 23 (21.1) | 8 (22.2) | 1.07  [0.41, 2.58] | 0.887 |
| Chronic obstructive pulmonary disease (%) | 10 (6.8) | 6 (5.5) | 4 (11.1) | 2.15  [0.52, 7.99] | 0.259 |
| Chronic kidney disease (%) | 15 (10.3) | 13 (11.9) | 2 (5.6) | 0.43  [0.07, 1.68 | 0.288 |
| Cancer (%) | 16 (11.0) | 13 (11.9) | 3 (8.3) | 0.67  [0.15, 2.24 | 0.553 |
| More than seven days of symptoms (%) | 80 (55.6) | 62 (57.9) | 18 (50.0) | 0.73  [0.34, 1.55] | 0.407 |
| Dyspnea (%) | 78 (53.4) | 49 (45.0) | 28 (77.8) | 4.29  [1.86, 10.85] | 0.001 |
| Confusion (%) | 7 (4.8) | 3 (2.8) | 4 (11.1) | 4.42  [0.93, 23.41] | 0.06 |
| Temperature > 38.2 °C (%) | 81 (56.6) | 56 (52.3) | 25 (69.4) | 2.07  [0.94, 4.77] | 0.076 |
| Heart rate > 100 bpm (%) | 48 (33.6) | 32 (29.9) | 16 (44.4) | 1.87 [0.86, 4.08] | 0.113 |
| Respiratory rate > 22/min (%) | 83 (58.0) | 50 (47.2) | 32 (88.9) | 8.96  [3.27, 31.66] | <0.001 |
| Systolic blood pressure < 100 mmHg (%) | 26 (18.1) | 13 (12.1) | 13 (36.1) | 4.09 [1.67, 10.11] | 0.002 |
| NEWS ≥ 7 (%) | 60 (43.5) | 34 (32.1) | 26 (81.2) | 9.18 [3.66, 26.55] | <0.001 |
| qSOFA score ≥ 2 (%) | 23 (16.1) | 9 (8.5) | 14 (38.9) | 6.86  [2.68, 18.47] | <0.001 |
| CRB-65 score ≥ 2 (%) | 40 (28.6) | 20 (19.4) | 19 (52.8) | 4.64 [2.07, 10.65] | <0.001 |
| Any radiological infiltrate (%) | 101 (69.2) | 66 (60.6) | 34 (94.4) | 11.0  [3.14, 70.45] | 0.001 |
| Bilateral radiological infiltrate (%) | 83 (56.8) | 51 (46.8) | 31 (86.1) | 7.05  [2.75, 21.87] | <0.001 |
| Acute kidney injury on admission (%) | 49 (45.0) | 18 (17.3) | 17 (47.2) | 4.27 [1.87, 9.91] | 0.001 |
| D-dimer ≥ 1000 ng/ml (%) | 36 (25.5) | 29 (36.7) | 19 (65.5) | 3.28  [1.37, 8.25] | 0.009 |
| C-reactive protein ≥ 40 mg/L (%) | 86 (63.7) | 53 (54.1) | 32 (88.9) | 6.79  [2.46, 24.09] | 0.001 |
| Procalcitonin ≥ 0.5 µg/l (%) | 11 (11.8) | 3 (4.8) | 7 (23.3) | 5.99  [1.52, 29.66] | 0.015 |
| Ferritin ≥ 300 µg/l (%) | 44 (51.2) | 53 (86.9) | 22 (91.7) | 1.66  [0.38, 11.59] | 0.541 |
